# Evolution of a Functional Head Joint in Deep-Sea Fishes (Stomiidae)

**DOI:** 10.1371/journal.pone.0170224

**Published:** 2017-02-01

**Authors:** Nalani K. Schnell, G. David Johnson

**Affiliations:** 1 Institut de Systématique, Évolution, Biodiversité. ISYEB–UMR 7205 –CNRS–MNHN–UPMC–EPHE. Muséum national d’Histoire naturelle, Sorbonne Universités, Paris, France; 2 Department of Vertebrate Zoology, National Museum of Natural History, Smithsonian Institution, Washington, DC, United States of America; University of Kansas, UNITED STATES

## Abstract

The head and anterior trunk region of most actinopterygian fishes is stiffened as, uniquely within vertebrates, the pectoral girdles have a direct and often strong connection through the posttemporal to the posterior region of the skull. Members of the mesopelagic fish family Stomiidae have their pectoral girdle separated from the skull. This connection is lost in several teleost groups, but the stomiids have an additional evolutionary novelty—a flexible connection between the occiput and the first vertebra, where only the notochord persists. Several studies suggested that stomiids engulf significantly large prey items and conjectured about the functional role of the anterior part of the vertebral column; however, there has been no precise anatomical description of this complex. Here we describe a unique configuration comprising the occiput and the notochordal sheath in *Aristostomias*, *Eustomias*, *Malacosteus*, *Pachystomias*, and *Photostomias* that represents a true functional head joint in teleosts and discuss its potential phylogenetic implications. In these genera, the chordal sheath is folded inward ventrally beneath its connection to the basioccipital and embraces the occipital condyle when in a resting position. In the resting position (wherein the head is not manipulatively elevated), this condyle is completely embraced by the ventral fold of the notochord. A manual manipulative elevation of the head in cleared and stained specimens unfolds the ventral sheath of the notochord. As a consequence, the cranium can be pulled up and back significantly farther than in all other teleost taxa that lack such a functional head joint and thereby can reach mouth gapes up to 120°.

## Introduction

"A rubber neck is a necessary part of equipment."                                    **Peter O'Toole**

**Fig 1:**
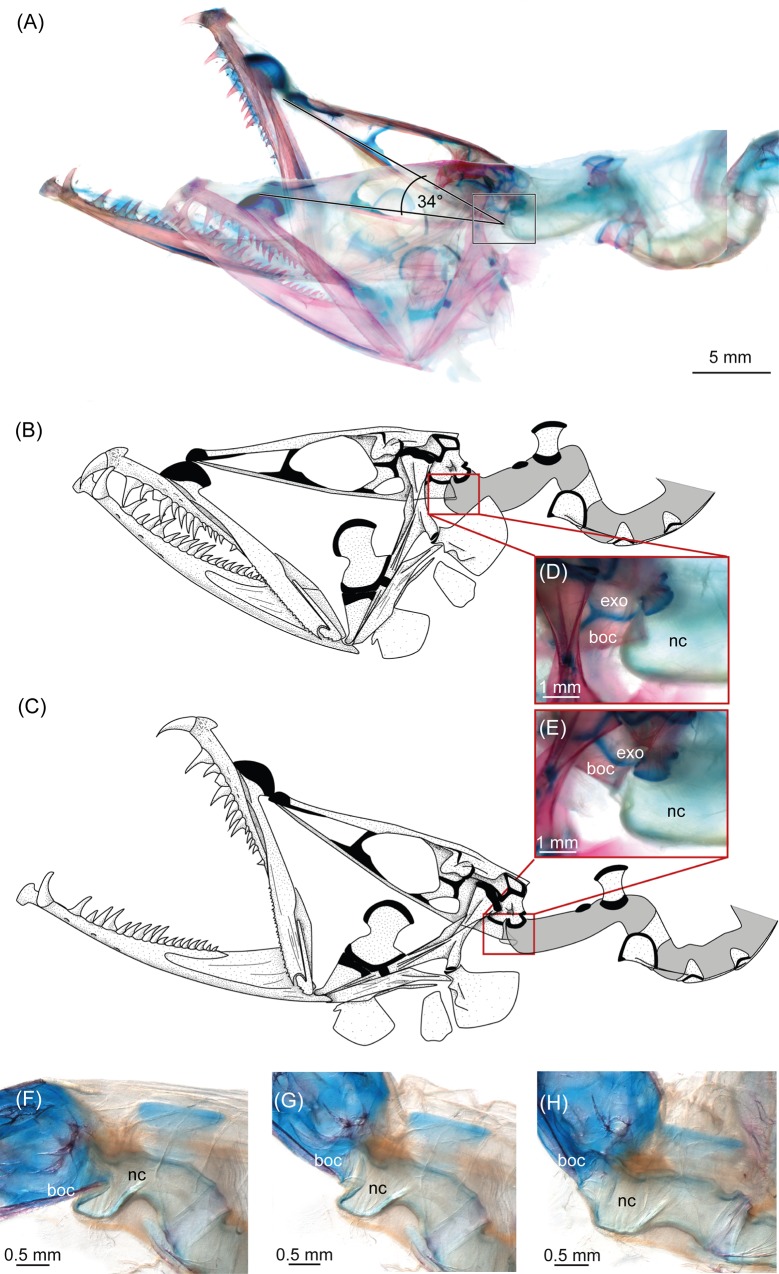
Head in “resting position” (mouth closed) and during manipulative elevation (mouth open), showing the functional head joint in *Eustomias obscurus*, USNM 206711 (A-E), and *Eustomias macronema*, BMNH 2007.10.31.12 (F-H). (A) Cleared and stained (c&s) specimen with manipulative elevation. (B) Drawing of the resting position and (C) manipulative elevation. (D) Zoom onto the functional head joint in a c&s specimen during resting position and (E) manipulative elevation in a c&s specimen. (F-H) Zoom onto the functional head joint in a c&s specimen during (E) resting position and (G-H) manipulative elevation. boc, basioccipital; exo, exoccipital; nc, notochord.

**Fig 2:**
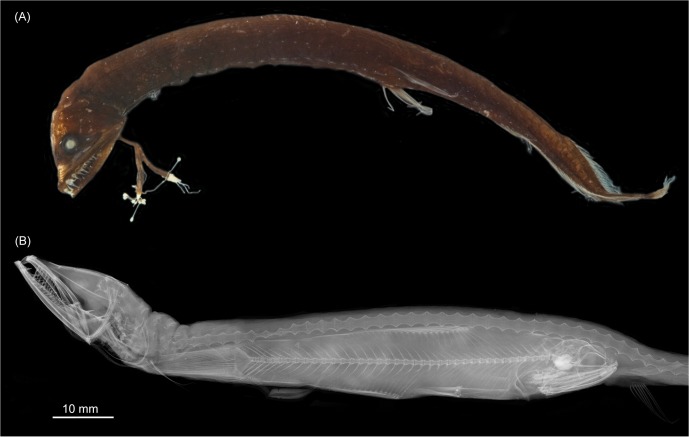
External and internal morphology of *Eustomias* spp. (A) *Eustomias fissibarbis*, USNM 270587. (B) X-ray of *Eustomias obscurus*, USNM 206711 showing a large, ingested myctophid.

The family Stomiidae represents one of the dominant fish families in mesopelagic ecosystems [1], exhibiting an array of specializations to a predatory existence in this environment, e.g. huge mouth gapes with prominent teeth, distensible stomachs, elongated dark bodies with photophores, and chin barbels with bioluminescent tissue [2–8]. *Malacosteus*, *Aristostomias*, and *Photostomias* lack an intermandibular membrane [3], and as a consequence jaw-closing velocity is increased [3,9]. *Malacosteus*, *Aristostomias* and *Pachystomias* possess far-red emitting photophores and a visual system that is sensitive to such long-wave emissions [10,11]. Some stomiid taxa have from one to ten anterior vertebrae reduced or entirely absent [2,12,13], and all of them have a distinctive gap between the occiput and the first vertebra, wherein only the flexible notochord persists [13,14]. This occipito-vertebral gap does not result from loss or reduction of vertebrae, but rather from an elongation of the notochord in this area of the vertebral column [13,14]. The unparalleled occipito-vertebral gap in stomiids allows a considerable degree of cranial elevation and is notably enhanced in the genera that share the functional head joint described herein (Fig 1A). We propose that this feature is an adaptation to extend the reach of the mouth gape antero-dorsally and for engulfing particularly large prey items. The separation of the pectoral girdle from the skull, due to the loss of the posttemporal and extrascapular [15] in all genera herein discussed, reinforces the maneuverability of the head. Stomach content analyses support this hypothesis, reporting that *Aristostomias*, *Eustomias* (Fig 2), and *Pachystomias* are piscivorous, feeding mainly on myctophids [6,8]. Only *Malacosteus* and *Photostomias* were found to feed mainly on crustaceans (copepods and penaeidean shrimps) [6,8]. It has been suggested that there has been a secondary reversion to planktivory in *Malacosteus* [8] that is correlated with the unique characters of red vision and red bioluminescence [10,11,16].

Our objectives in this study are to describe the anatomy of the unique functional head joint within several stomiid genera, to test its homology, and to see if this complex morphological character corroborates the most recent molecular phylogeny of the family [16].

## Materials and Methods

Our research employed only ethanol-preserved specimens deposited in museum collections and did not involve animal experimentation or examination of fossil specimens. Examined material is listed in the following section “Material examined” and is deposited in the following institutions: National Museum of Natural History, Smithsonian Institution, USA (USNM); Scripps Institution of Oceanography, USA (SIO); Museum of Comparative Zoology, Harvard University, USA (MCZ); British Museum of Natural History, UK (BMNH); National Museum of Nature and Science, Japan (NSMT-P); Virginia Institute of Marine Science, USA (VIMS). Access to material of those collections was authorized by respective curators and specimens were examined at their original institutions or loaned to the Muséum national d’Histoire naturelle (MNHN). No permits were required for the described study, which complied with all relevant regulations. Preserved specimens were cleared and double stained (c&s) [17,18] and afterwards examined and dissected using a Zeiss Discovery V12/V20 stereomicroscope. One specimen was cleared and triple stained (c&ts) with Sudan Black B, in order to stain nerves, following the alcian blue-alizarin red staining [19]. Photographs were taken with an attached Axiocam microscope camera and processed with the Zeiss AxioVision or ZEN software to obtain composite images with an increased depth of field. The specimens for the histological serial sections (hss) were embedded in paraffin and stained with Azan [20]. All measurements referenced herein are standard length, SL, unless otherwise mentioned.

### Material examined

***Aristostomias xenostoma***, USNM 296715, 3 specimens, 123 mm, 140 mm, including 1 c&s, 83 mm; ***Bathophilus* sp**., USNM 325530, 1 specimen c&s, 73 mm; ***Bathophilus filifer***, SIO 03–189 1 specimen (hss), 80 mm; SIO 76–42, 1 specimen c&s, 75 mm TL; ***Bathophilus vaillanti***, USNM 234150, 1 specimen c&s, 101 mm; ***Eustomias* sp**., MCZ 62637, 4 specimens c&s, 26–45 mm (26, 30, 32, 45 mm); USNM 394242, 1 specimen c&s, 59 mm; ***Eustomias bifilis***, SIO 97–89, 2 specimens, 108 mm TL; including 1 c&s, 105 mm TL; ***Eustomias filifer***, BMNH 2007.10.31.64, 1 specimen (hss), 97 mm TL; ***Eustomias fissibarbis***, USNM 270587, 1 specimen, 120 mm; ***Eustomias macronema***, BMNH 2007.10.31.12, 1 specimen c&s, 65 mm TL; ***Eustomias obscurus***, USNM 206711, 5 specimens, 131–199 mm, including 1 c&s, 199 mm, and 1 c&ts, 147 mm; (131, 135, 147, 179, 199 mm); USNM 234416, 1 specimen c&s, 71 mm; USNM 234444, 1 specimen c&s, 59 mm; ***Flagellostomias boureei***, BMNH 2002.8.5.786–788, 1 specimen c&s, 161 mm; ***Grammatostomias circularis***, NSMT-P 99317, 1 specimen (hss), 149 mm; ***Grammatostomias dentatus***, USNM 234036, 1 specimen c&s, 76 mm; VIMS 11846, 2 specimens, 117 mm, including 1 c&s, 111 mm; ***Idiacanthus antrostomus***, SIO 60–459, 2 specimens, 182 mm, including 1 c&s, 320 mm; SIO 70–237, 3 specimens c&s, 57–135 mm (57, 75, 135 mm); SIO 97–85, 1 specimen (hss), 310 mm; ***Malacosteus australis***, USNM 296675, 1 specimen c&s, 110 mm; ***Malacosteus niger***, SIO 73–25, 3 specimens, 135 mm, including hss, 100 mm, and 1 c&s, 130 mm; USNM 296813, 1 specimen c&s, 74 mm; ***Pachystomias microdon***, USNM 297922, 1 specimen c&s, 168 mm; USNM 297923, 1 specimen c&stained with alizarin, 156 mm; ***Photostomias* sp**., USNM 296650, 1 specimen c&s, 92 mm; ***Photostomias guernei***, BMNH 2007.10.31.6, 1 specimen c&s, 50 mm; BMNH 2007.10.31.19, 1 specimen c&s, 112 mm.

## Results

**Fig 3:**
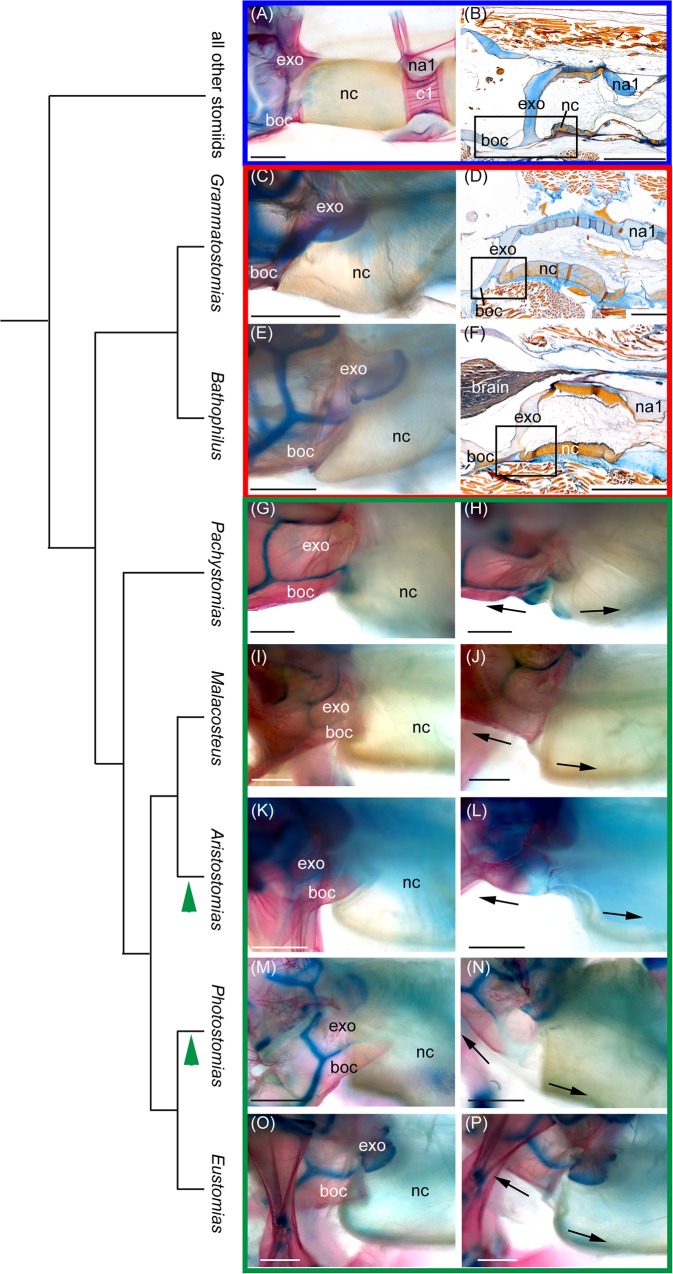
Presence of the functional head joint mapped onto the latest molecular analysis [15] of the family Stomiidae. (A, B) Functional head joint absent in basal stomiids (blue box). The notochord does not overlap the basioccipital ventrally (small black box), e.g., in the cleared and double stained (c&s) specimen of (A) *Flagellostomias boureei*, BMNH 2002.8.5.786–788 and the histological sagittal section (hss) of (B) *Idiacanthus antrostomus*, SIO 97–85. (C–F) Intermediate stage of the functional head joint (red box), where the chordal sheath slightly overlaps the basioccipital ventrally (best seen in the histological sections (D, F). (C) *Grammatostomias dentatus*, USNM 234036 (c&s). (D) *Grammatostomias circularis*, NSMT-P 99317 (hss) and (E) *Bathophilus vaillanti*, USNM 234150 (c&s). (F) *Bathophilus filifer*, SIO 03–189 (hss). (G–P) Presence of the functional head joint (green box). The notochord overlaps the basioccipital ventrally in a resting position (G, I, K, M, O). Unfolding of the ventral sheath of the notochord during manipulative elevation of the head in c&s specimens (H, J, L, N, P). Green arrow heads indicate the two genera (*Aristostomias* and *Photostomias*) that have the occipital condyle composed of the exoccipitals and the basioccipital. (G) *Pachystomias microdon*, USNM 297922. (H) *Malacosteus australis*, USNM 296675. (I) *Aristostomias xenostoma*, USNM 296715. (J) *Photostomias* sp., USNM 296650. (K) *Eustomias obscurus*, USNM 206711. boc, basioccipital; c1, first centrum; exo, exoccipital; na1, first neural arch; nc, notochord. Scale bars, 1 mm.

In basal stomiid genera, the notochordal sheath forms a straight connection between the occiput (the basioccipital and exoccipital) and the first vertebra, e.g., in *Flagellostomias boureei* (Fig 3A) and *Idiacanthus antrostomus* (Fig 3B). Whereas in *Aristostomias*, *Eustomias*, *Malacosteus*, *Pachystomias*, and *Photostomias* (Fig 3G, 3I, 3K, 3M and 3O) the notochord is folded inward ventrally beneath its connection to the basioccipital, and this extra sheath embraces the occipital condyle in a resting position (i.e., wherein the head is not manually elevated and the jaws are closed, Fig 1B, 1D and 1F). The occipital condyle is formed by only the posteriorly elongated basioccipital in *Eustomias*, *Malacosteus*, and *Pachystomias*, but in *Aristostomias* and *Photostomias*, it also includes the exoccipitals. A manipulative opening of the mouth, and resultant elevation of the head in cleared and stained specimens, unfolds the ventral sheath of the notochord (Figs 1C, 1E, 1G, 1H, 3H, 3J, 3L, 3N and 3P). As a consequence, the cranium can be pulled up and back farther than in taxa that lack such a functional head joint. Manipulative elevation of the neurocranium results in a 30° (*Eustomias*, *Grammatostomias*, *Pachystomias*) to about 80° (*Aristostomias*, *Malacosteus*, *Photostomias*) dorsal flexion. At the same time, the jaws are rotated anteriorly. When manipulative elevation of the head is terminated, the ventral sheath of the notochord folds back inward to its original position embracing the occipital condyle (Fig 1B, 1D and 1F), and the neurocranium moves back into a resting position, where it is ventrally declined in *Aristostomias*, *Malacosteus*, and *Photostomias* [3]. In *Bathophilus* and *Grammatostomias*, the ventral sheath of the notochord overlaps the basioccipital posteriorly to a much lesser extent, and the posterior margin of the basioccipital forms a ventrally directed rim to which the notochordal sheath attaches (Fig 3C–3F). Histology shows that anlagen for the head joint are present in *Bathophilus* and *Grammatostomias*, wherein the chordal sheath slightly overlaps the basioccipital ventrally and thus seemingly represents an intermediate stage (red box in Fig 3).

## Discussion

Cranial elevation driven by the epaxial musculature is a common feature of feeding in fishes, as it contributes to mouth opening and promotes jaw protrusion [21]. In stomiids, the presence of considerable flexibility in the anterior part of the vertebral column coincident with a functional head joint allows extension of the mouth gape antero-dorsally and enables these taxa to have gapes up to 120° [3, 9].

**Fig 4:**
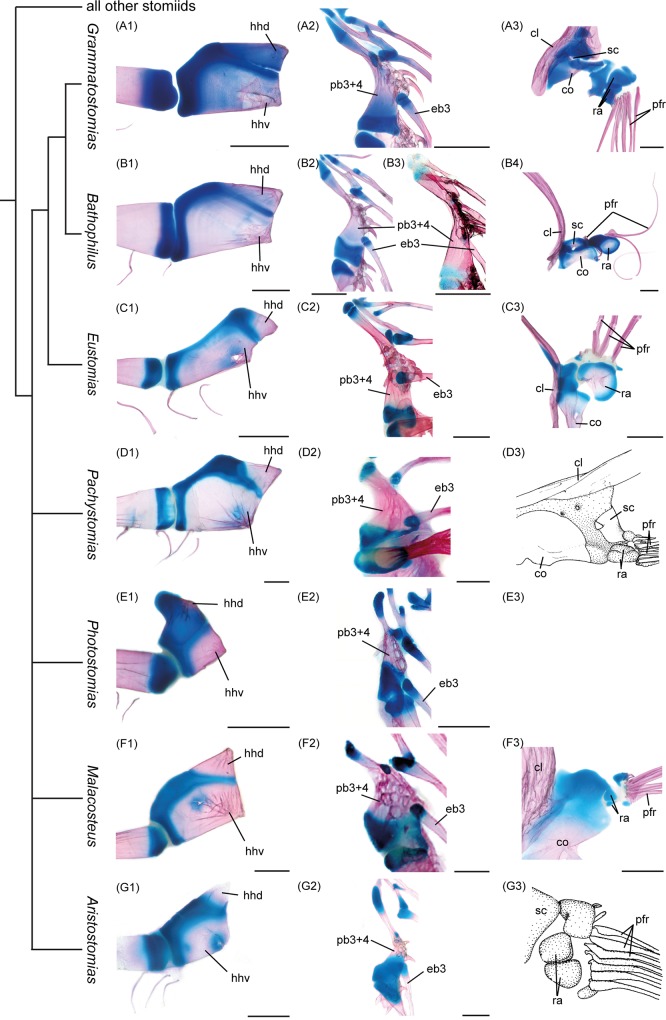
Illustration of Fink’s [15] characters placing *Eustomias* as sister to *Bathophilus*+*Grammatostomias*. Cladogram according to Fink [15]; left column, compound hypohyal; middle column, articulation of third epibranchial with fused third and fourth pharyngobranchials of dorsal gill arches; right column, bases of pectoral fin rays. All images show c&s specimens, except (D3) and (G3), which are modified drawings from Fink [14]. (A) *Grammatostomias dentatus*, USNM 234036. (B1,2,4) *Bathophilus vaillanti*, USNM 234150. (B3) *Bathophilus filifer*, SIO 76–42. (C) *Eustomias obscurus*, USNM 206711. (D) *Pachystomias microdon*, USNM 297922. (E) *Photostomias* sp., USNM 296650. *Photostomias* lacks pectoral fin rays. (F) *Malacosteus australis*, USNM 296675. (G) *Aristostomias xenostoma*, USNM 296715. cl, cleithrum; co, coracoid; eb3, third epibranchial, hhd, dorsal hypohyal; hhv, ventral hypohyal; ra, radial; sc, scapula; pb3-4, fused pharyngobranchials 3+4; pfr, pectoral fin ray. Scale bars, 1 mm.

Although evidence has been provided that the previously recognized six stomiatoid families (Astronesthidae, Chauliodontidae, Idiacanthidae, Melanostomiidae, Malacosteidae, and Stomiidae) should be placed in a single, expanded Stomiidae [15], these families are sometimes still recognized as separate subfamilies [22,23]. The presence of a functional head joint in *Aristostomias*, *Eustomias*, *Malacosteus*, *Pachystomias*, and *Photostomias* renders two of those putative subfamilies, Malacosteinae and Melanostomiinae, paraphyletic. The phylogenetic hypothesis inferred by this character corresponds (with one difference) with the latest analysis [16] based on rod opsin and three other nuclear gene fragments (rag1, myh6, enc1). The formation of the occipital condyle by the basioccipital and the exoccipital in *Photostomias* and *Aristostomias* suggests a sister relationship between the two taxa, contrary to the relationship proposed in the latest anaylsis [16], wherein *Aristostomias* is sister to *Malacosteus* based on the four nuclear loci, their red sensitivity, and photophore emission, exceeding 700 nm. We mapped the presence of the functional head joint on this previous analysis [16] of the family (Fig 3). It’s presence corroborates the placement of *Eustomias* within the “malacosteine” loosejaw + *Pachystomias* clade, with *Bathophilus* + *Grammatostomias* sister to this clade. *Eustomias* has traditionally been placed somewhere within the “Melanostomiinae,” and in his extensive morphological treatise on 25 stomiid genera Fink [15] placed it for the first time as sister to *Bathophilus* + *Grammatostomias* based on three osteological characters. Our analysis as well as the recent molecular analysis [16] places *Eustomias* within the “Malacosteinae.” Accordingly, below we discuss the three characters that led to Fink’s [15] alternative placement within the “melanostomiines” (Fig 4).

1. In reference to the hypohyal complex (Fig 4, left column): “In *Bathophilus*, *Eustomias*, and *Grammatostomias*, the hypohyal element is about twice as long anteroposteriorly as it is dorsoventrally, and its longest dorsoventral distance is posterior to the anterior border of the element” (p. 44 [15]). We agree that the compound hypohyals (comprising dorsal and ventral elements) are more elongate in these three genera relative to those of *Pachystomias*, *Malacosteus*, *Aristostomias*, and *Photostomias*. Those of *Bathophilus* and *Grammatostomias* are quite similar in size and shape, but differ from that of *Eustomias* in which the dorsal hypohyal lies anterior to the extremely elongate ventral element rather than dorsal to it as in *Bathophilus* and *Grammatostomias* and all other “Malacosteinae” + *Pachystomias* (Fig 4, left column). In terms of character information we consider the relative positions of the anterior and posterior hypohyals to one another equally or more relevant than the length to depth ratio of the compound element.
2. In reference to the dorsal gill arches (Fig 4, middle column): “In *Bathophilus*, *Eustomias*, and *Grammatostomias*, the third epibranchial articulates with the third pharyngobranchial at a point anterior to the posterior border of the pharyngobranchial ossification. (…) In other stomiids, the third epibranchial articulates with the third pharyngobranchial adjacent to the posterior border of the pharyngobranchial ossification” (p. 51 [15]). *Bathophilus* and *Grammatostomias* are very similar regarding the shape of the fused third and fourth pharyngobranchial (pb3+pb4) [24] and, in fact, in overall configuration of the entire dorsal complex. The third epibranchial (eb3) articulates with the fused pb3+pb4 slightly anterior to the cartilaginous posterior area. Shape of the fused pb3+pb4, articulation of the third epibranchial, and overall configuration of the dorsal complex is similar in *Aristostomias*. In *Eustomias* the fused pb3+pb4 element is extremely elongate, and the articulation of the third epibranchial is even farther anterior than in *Bathophilus* and *Grammatostomias*. We also note that the point of articulation of the third epibranchial can be intraspecifically variable. In *Bathophilus filifer* (Fig 4B3) the point of articulation is as far anterior as in *Eustomias obscurus* (Fig 4C2) and therewith different from the condition in *Bathophilus vaillanti* (Fig 4B2). We agree that the more anterior articulation of the third epibranchial could be construed as a synapomorphy of the latter three genera, as Fink proposed.
3. In reference to pectoral-fin rays (Fig 4, right column): “In *Bathophilus* (…), *Grammatostomias*, and *Eustomias* (…), the flanges for muscle attachment on the dorsal halves of the non-rod ray fin rays are greatly reduced in breadth (i.e., in the axis perpendicular to the axis of the rays). (…). In other stomiids, such flanges are more developed” (p. 80 [15]). We agree that there are no such flanges in *Eustomias* and *Grammatostomias*, although in the latter the first ray has a little broadening at the base. *Bathophilus* has two separated sets of fin rays. The two smaller ones articulating at the scapula have slightly broadened bases but no obvious flanges. However, the ray attaching to the radial has an obvious triangular base that we would call a flange. Furthermore, the suggested flanges in *Pachystomias* are no more developed than they are in *Grammatostomias*. Again, we see no clear character in this complex to support a close relationship between *Bathophilus*, *Grammatostomias*, and *Eustomias*.

There is no doubt about the sister group relationship between *Bathophilus* and *Grammatostomias* based on these three characters, but we maintain that the hyoid and pectoral fin ray characters are open to alternative interpretations and thus do not unequivocally support a relationship between *Eustomias*+(*Bathophilus*+*Grammatostomias*). Fink’s character of the dorsal gill arches may be valid, but that complex and others need to be more comprehensively investigated to fully understand and support a specific placement of *Eustomias* within the “Malacosteinae,” particularly in light of our documentation of the distribution of the functional head joint among these taxa.

In an anatomical and functional morphology study [3] on the jaw apparatus of two loosejaw genera, published in the 19th century in German, it was noted that the connection between the occiput and the notochord in *Photostomias* and *Malacosteus* resembles a ball and socket joint, but the details of this specialized connection could not be fully understood with the rigid, ethanol preserved specimens those authors had available.

An occipito-vertebral gap between the first and second vertebrae and intervertebral spaces is documented elsewhere only for the lepidogalaxiid, *Lepidogalaxias salamandroides*, and here, smaller gaps are present between all succeeding vertebrae [25]. Observations of a live specimen proved that this facilitates movements of the head, e.g. elevating and bending the neck to each side [25]. For barbeled dragonfishes (family Stomiidae), the very few live records do not include feeding activity, and such observations are unlikely to be available as long as specimens cannot be kept alive, but such observations might prove invaluable in delivering a stable hypothesis of the functional significance of this exceptional vertebrate head joint.
